# Mitochondrial genome of the critically Endangered silver boa (*Chilabothrus argentum*; Squamata: Boidae)

**DOI:** 10.1080/23802359.2022.2089067

**Published:** 2022-06-28

**Authors:** Alyssa A. Vanerelli, Aryeh H. Miller, L. Caden Comsa, Anthony J. Geneva, R. Graham Reynolds

**Affiliations:** a Center for Computational and Integrative Biology, Rutgers University–Camden, Camden, NJ, USA; b Department of Biology, Washington University in St. Louis, St. Louis, MO, USA; c Department of Biology, Rutgers University–Camden, Camden, NJ, USA; d Department of Biology, University of North Carolina Asheville, Asheville, NC, USA

**Keywords:** Caribbean, Illumina, mitogenome, next-generation sequencing, phylogeny

## Abstract

We present the complete mitochondrial genome of *Chilabothrus argentum*, which is 17,345 bp in length, has 22 transfer ribonucleic acids (tRNAs), 2 ribosomal subunits (rRNAs), 13 protein-coding genes, an origin of the light-strand replication (O_L_), and two control regions (CR1, CR2). A maximum likelihood phylogenetic estimate using nine other snake mitochondrial genomes yields agreement with previous investigations into the evolutionary relationships of snakes.

The Silver Boa (*Chilabothrus argentum* Reynolds 2016) is endemic to the Conception Island Bank in the Bahamas (Reynolds et al. 2016). With a population size of only 135 ± 35 individuals occurring in an area of habitat <0.5 km^2^ (Reynolds et al. in review), this species is listed as Critically Endangered on the International Union for the Conservation of Nature (IUCN) Red List (Reynolds 2017) and is close to extinction. Consequently, rapidly developing genomic resources to characterize genetic diversity within the sole population of this species is crucial to aiding ongoing conservation efforts. Here we describe the complete mitochondrial genome of *C. argentum* using mitochondrial bycatch from ultraconserved elements sequencing and whole genome sequencing.

We obtained a tissue sample from an individual captured by hand (23°50.3′N, 75°6.9′W), which was given voucher number RGRCB047 and stored in the vertebrate collection of The University of North Carolina, USA (The person in charge of the collection: RG Reynolds; email: greynold@unca.edu). We followed methods detailed by Miller et al. (2019) to extract, sequence, and assemble the partial mitochondrial genome (GenBank ID MW176073). The resulting assembly included all protein coding genes but lacked portions of the control region. To generate a complete, circular assembly, we then selected a single individual for whole genome sequencing (WGS). Using the Wizard SV^®^ Kit (Promega, Madison, WI, USA), we extracted whole genomic DNA and generated a next generation sequencing library using a KAPA HyperPlus prep kit (Roche, Basel, Switzerland). We sequenced the library on an Illumina NovaSeq 6000 instrument at the Genomic Center, Rutgers New Jersey Medical School using paired end 2 × 150bp chemistry. We trimmed raw WGS reads using Trimmomatic v0.39 (Bolger et al. 2014) and used ILLUMINACLIP to remove sequencing adapters. We removed nucleotides with quality scores below 20 from the leading and trailing ends of each read. We truncated reads from the ends if sliding windows of 13 bp had an average quality score less than 20. Then, for reads less than 23 bp, we removed that read and its paired read. We used NOVOPlasty 4.3.1 (Dierckxsens et al. 2017), a seed-extend based assembler optimized for circular plastid genomes, to perform a circularized assembly. We used our initial partial genome assembly as the seed and the reference, and the trimmed reads as input in NOVOPlasty. We used the MITOS and MITOS2 (Bernt et al. 2013) webservers (http://mitos.bioinf.uni-leipzig.de/index.py; http://mitos2.bioinf.uni-leipzig.de/index.py) to annotate the mitogenome, then manually verified and adjusted the annotations as needed with comparison to a reference sequence of *B. constrictor* (GenBank ID AB177354; Dong and Kumazawa 2005) in Geneious Prime 2022.0.2 (https://www.geneious.com).

We aligned the final *C. argentum* mitogenome with all available boid mitogenomes from GenBank using the MUSCLE v3 algorithm (Edgar 2004) in Geneious Prime. We performed a maximum likelihood analysis on the 13 concatenated protein coding regions (11,340 bp total) using the RaxML v8.2.9 (Stamatakis 2014) plugin in Geneious Prime with a GTR GAMMA model and rapid bootstrap inferences (1000 replicates) followed by a thorough ML search. We rooted the tree with *Acrochordus granulatus* (GenBank ID AB177879; Dong and Kumazawa 2005) in FigTree v1.4.4.

The assembled mitochondrial genome for *C. argentum* (GenBank ID ON015858) is 17,345 bp in length, comparably shorter than a previously published *B. constrictor* mitogenome (18,905 bp; Dong and Kumazawa 2005). Nucleotide composition in the *C. argentum* assembly is 36.4% A’s (6,308 bp), 24.9% C’s (4,327 bp), 12.3% G’s (2,127 bp), and 26.4% T’s (4,583). GC content in the mitogenome of *C. argentum* (37.2%) is nearly identical to that of *B. constrictor* (38.5%). We recovered no deviations from mitogenome gene composition or order compared to that of *B. constrictor*, with 22 transfer ribonucleic acids (tRNAs), 2 ribosomal subunits (rRNAs), 13 protein-coding genes, an origin of the light-strand replication (O_L_), and two control regions (CR1, CR2). The O_L_ is 33 bp in length and located within the WANCY tRNA cluster, between tRNA^Asn^ and tRNA^Cys^. CR2 is a 1,079 bp segment between tRNA^Ile^ and tRNA^Leu^, and includes several identical stretches shared with the longer CR1 (982 bp).

Maximum likelihood phylogenetic analysis (Figure 1) yielded a phylogeny congruent with previous studies investigating the evolutionary relationships of Alethinophidian snakes (Douglas and Gower 2010). Our analysis inferred *C. argentum* to be sister to *B. constrictor* with moderate support and strong support for the monophyly of a clade consisting of those two species and *Eryx tataricus*. These relationships are expected given previous phylogenetic analyses using other molecular markers (Reynolds et al. 2018).

**Figure 1.**
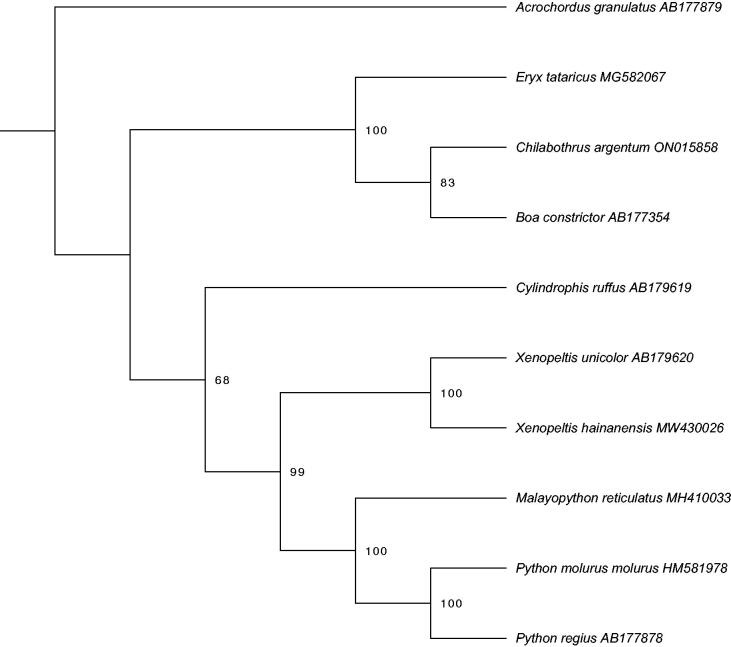
Maximum likelihood phylogeny of aligned concatenated protein-coding loci (11,340 bp) with 10 serpent species. GenBank accession numbers are indicated on tip labels. Numbers at nodes represent bootstrap support.

## Ethical approval

We obtained research and collection permits from the Bahamas Environment, Science and Technology (B.E.S.T.) Commission, the Bahamas National Trust, and the Ministry of the Environment. We exported the samples under the Department of Agriculture CITES permits 2015/196 and 2017/295 from the Commonwealth of the Bahamas. All animal capture, handling, and sampling was performed following the American Society of Ichthyologists and Herpetologists (ASIH) guidelines for use of reptiles and amphibians in research and all methods were approved under the authors’ IACUC permits.

## Author contributions

AAV, AHM, and RGR were involved in the conception and design; AAV, AHM, AJG, LCC, and RGR were involved in analysis and interpretation of the data; AAV, AHM, and RGR were involved in the drafting of the paper; AAV, AHM, AJG, LCC,and RGR were involved in revising it critically for intellectual content; all authors gave their final approval of the version to be published; and all authors agree to be accountable for all aspects of the work.

## Data Availability

The genome sequence data from this study are openly available in GenBank of NCBI at (https://www.ncbi.nlm.nih.gov/) under the accession no. ON015858. The associated Bio-Project, SRA, and Bio-Sample numbers are PRJNA835855, SRR19127218, and SAMN28117297 respectively.
